# Collagen Matrix Versus Subepithelial Connective Tissue for Recession Coverage: A Systematic Review

**DOI:** 10.1111/odi.15203

**Published:** 2024-12-09

**Authors:** Alessandro Zangani, Miriana Gualtieri, Alessia Pardo, Annarita Signoriello, Paolo Faccioni, Gianluca Colapinto, Funda Goker, Giorgio Lombardo, Massimo Del Fabbro, Massimo Albanese

**Affiliations:** ^1^ Dentistry and Maxillofacial Surgery Unit, Department of Surgery, Dentistry, Paediatrics and Gynaecology (DIPSCOMI) University of Verona Verona Italy; ^2^ Department of Biomedical, Surgical and Dental Sciences Università Degli Studi di Milano Milan Italy; ^3^ Fondazione IRCCS Ca’ Granda Ospedale Maggiore Policlinico Milan Italy

**Keywords:** collagen matrix, connective tissue, gingival recession, periodontal surgery, periodontology

## Abstract

**Objective:**

To evaluate the outcome of collagen matrix (CMX) compared with subepithelial connective tissue graft (SCTG) in gingival recession coverage (RC) surgery.

**Methods:**

Review protocol was registered in PROSPERO. The search was conducted on MEDLINE, Cochrane Library, and Scopus databases. Randomized studies comparing CMX versus. SCTG or CMX versus. covering procedures without any filling material, for class I recession treatment were included. Risk of bias assessment and quantitative analysis were performed.

**Results:**

Of 168 records, 11 randomized clinical trials were included. The meta‐analysis revealed no statistically significant difference in terms of %RC (*p* = 0.37); there was a statistically significant difference in terms of recession reduction (*p* = 0.02) and keratinized tissue width (*p* = 0.03) in favor of SCTG cases. CMX showed a statistically significantly better result compared to no grafting, regarding %RC (*p* = 0.003) and keratinized tissue thickness (*p* < 0.0001). The duration of the intervention was significantly shorter for CMX than for SCTG (*p* < 0.0001).

**Conclusion:**

CMX can be considered a viable material, especially when a ΔKTt increase and a reduction of intervention duration is needed. The indications for the choice, however, may depend on the individual local condition.

**PROSPERO Registration:** Registration in PROSPERO (International prospective register of systematic reviews): CRD42024555443

## 1 Introduction

Gingival recession (GR) is a common periodontal condition defined as the apical shifting of the gingival margin in relation to its physiological position, located 1–2 mm coronally to the cemento‐enamel junction (CEJ) (Cortellini and Bissada 2018; Cairo et al. 2011).

The presence and extension of GR tend to increase with age: according to a former study (Kitchin 1941) 51.6% of the population had GR; 57.7% of people over the age of 40 had GR, but only 15.5% of patients between the age of 20 and 29 had GR.

Over the years, the prevalence of GR in young people changed: (Ervin 1944) observed that in a total of 1272 patients, 60% of the young patients (20 years) had GR; another study (Gorman 1967) reported 62% of GR in people between the ages of 16 and 25, 90% between the ages of 26 and 35, 92% between the ages of 36 and 42, and 100% between the ages of 46 and 86. A 100% of GR was found in a study evaluating a group of dental students (Muhlemann 1974).

Currently, GRs affect almost the entire population of the US. Data collected from 2009 to 2014 in the U.S. population showed that the patient‐level prevalence of mid‐buccal GRs in the 30–34 years' age group was 80%, in the 35–49 years' group was 90%, in the 50–64 years' group was 95% and, in the 65 + years group, it was 97% (Romandini et al. 2020).

Recently, a study in an Italian population (Romano et al. 2022) reported lower values of GRs than the aforementioned studies: 34% between 20 and 39 years, 61% between 40 and 59 years, 73% between 60 and 75 years.

During the 1920s and the 1930s, the etiology of GR was undoubtedly associated with occlusal trauma (Stillman 1921). Later, different authors refuted such statement and proposed a long series of possible causes of GR beyond occlusal trauma (Moscow 1965; Bernimoulin 1974; Hall 1984), such as alveolar bone dehiscence (Saminsky et al. 2022), thin gingival phenotype (Kim, Bassir, and Nguyen 2020), dental ectopic eruption and malpositioning (Gürbüz et al. 2023; Tomina et al. 2021), orthodontic treatment, especially in patients with thin phenotype (Wennström et al. 1987; Yared, Zenobio, and Pacheco 2006), subgingival margin placement of restoration (Ericsson and Lindhe 1984; Gracis et al. 2001; Günay et al. 2000; Pama‐Benfenati et al. 1986; Tal et al. 1989) and frenulum insertion near the cervical region of gingiva (Tait 1934; Placek, Skach, and Mrklas 1974).

Gingival recessions can lead to esthetic and functional issues, including dentin hypersensitivity, root caries, and tooth support loss (Cairo, Pagliaro, and Nieri 2008).

Root coverage procedures' final goal is the complete root coverage with excellent soft tissue integration with adjacent sites (Cairo et al. 2009). These techniques are known to improve patient esthetics and reduce root hypersensitivity (Nieri et al. 2013).

Some of the most commonly used techniques to treat GR are the Coronally Advanced Flap (CAF) or modified Coronally Advanced Flap (mCAF) with or without subepithelial connective tissue graft (sCTG), and tunnel techniques such as the Coronally Advanced Tunnel technique (CAT) or modified Coronally Advanced Tunnel (mCAT) with or without sCTG.

More recently, industrial research has aimed to create a product that can mimic the features and behavior of autogenous connective tissue to avoid harvesting from the palate. The acellular dermal matrix derived from human donors is associated with ethical problems and the risk of transmitting infectious diseases. For these reasons, a new Xenogeneic Collagen Matrix (CMX) of porcine origin, indicated for root coverage and other periodontal plastic surgery, has recently been introduced in the market (Sanz et al. 2009). Different CMXs are produced by different manufacturers, but those most commonly used in the clinical scenario are characterized by a bi‐layered structure of type I and III collagen without cross‐linking (De Santis et al. 2022). These substitutes showed promising results, low morbidity, and allowed for a significant shortening of the intervention duration (McGuire et al. 2022; Toledano, et al. 2020a; Toledano, et al. 2020b; Zuhr, Bäumer, and Hürzeler 2014; Moraschini et al. 2020; Toledano‐Osorio et al. 2022).

Mucograft (Geistlich Pharma AG, Wolhusen, Switzerland) is a CMX designed specifically to increase keratinized tissue around implants or teeth and for root coverage procedures.

It has been reported that the use of CMX in guided tissue regeneration (GTR) yields promising outcomes in terms of root coverage (Rotundo and Pini‐Prato 2012), gingival tissue thickness (Hadzik et al. 2023; Puzio et al. 2020), and clinical attachment levels (Navya and Rajasekar 2022; Imber et al. 2021).

### 1.1 Rationale

Currently, there is no consensus regarding the effectiveness of CMX in gingival recession management. Some studies reported positive outcomes with the use of CMX (Sanz et al. 2009; Fu, Su, and Wang 2012), meanwhile others have found no significant differences between CMX and other barrier membranes or surgical techniques (Alauddin et al. 2022).

### 1.2 Objectives

This systematic review aimed to assess the available evidence regarding the use of CMX in the treatment of gingival recession. This review specifically evaluated the efficacy and safety of CMX in terms of clinical and patient‐reported outcomes as well as its potential advantages and disadvantages compared to sCTG.

This review aimed to identify existing evidence and knowledge gaps that may inform future research in this field by synthesizing and analyzing the current literature.

## 2 Materials and Methods

This study was conducted in accordance with PRISMA statement guidelines (2020). This systematic review was conducted according to the population, intervention, comparison, and outcome (PICO) format. We analyzed clinical trials involving patients with at least one gingival recession classified as Miller I or II who were treated with CMX + CAF/mCAF/mCAT, sCTG + CAF/mCAF/mCAT, or CAF alone.

### 2.1 Focused Question

In patients with at least one Miller Class I or II gingival recession, CMX was more effective in CAF/mCAF/mCAT as compared to sCTG in CAF/mCAF/mCAT or CAF alone in terms of clinical and patient‐reported outcome measures.

### 2.2 Eligibility Criteria

The following inclusion and exclusion criteria were applied to select the study:

#### 2.2.1 Inclusion Criteria

- Randomized controlled clinical trials (RCT) with a minimum follow‐up of 6 months;
- Date of publication starting 01/01/2013 up to the time of the last search 27/04/2023;
- Studies with ≥ 5 patients involved per group;
- Patients with single or multiple GRs classified as class I or II according to (Miller 1985) or class RT1 according to (Cairo et al. 2011);
- Studies comparing CMX versus sCTG in the surgical treatment of recession using CAF/mCAF/mCAT or CMX + CAF versus CAF alone.
- Human studies;
- Articles published exclusively in English.

#### 2.2.2 Exclusion Criteria

- Patients with single or multiple GRs are classified as class III or IV according to (Miller 1985) or class RT2 or RT3 according to (Cairo et al. 2011). These types of defects do not allow complete and predictable root coverage.
- Surgical interventions or biomaterials other than those previously specified.

### 2.3 Information Sources

Electronic searches were performed using MEDLINE (PubMed), Cochrane Library, and Scopus databases. The search was limited to studies published between January 2013 and April 2023.

### 2.4 Search Strategy

The electronic search was conducted by two independent examiners (AZ and MG) to minimize the reviewer bias.

The search on PubMed was searched using the following MeSH terms: ((((mucograft[All fields]) OR (collagen matrix[All fields]))) AND (gingival recession[All fields])) AND (connective tissue graft[All fields]).

The Cochrane Library search was conducted using the following terms: mucograft in All Text AND gingival recession in All Text AND connective tissue graft in All Text AND collagen matrix in All Text.

The search on Scopus was conducted using the following string: (TITLE‐ABS‐KEY (mucograft) OR TITLE‐ABS‐KEY (collagen AND matrix) AND TITLE‐ABS‐KEY (gingival AND recession) AND TITLE‐ABS‐KEY (connective AND tissue AND graft)).

### 2.5 Study Selection

Two independent examiners (AZ and MG) reviewed the titles and abstracts of the studies retrieved through the electronic search. In case of disagreement, the two reviewers analyzed the title and abstract jointly to arrive at a final decision concerning inclusion or exclusion. Articles identified as potentially helpful for answering the research question were considered eligible. The full text of the eligible studies was obtained and evaluated to check the compliance of the studies with the inclusion criteria. The reason for exclusion was recorded for all studies excluded at this stage.

### 2.6 Data Extraction

Data extraction was performed by filling in a table with the following data: author, publication year, study design, follow‐up, type of test surgery, type of control surgery, total number of patients, number of test patients, number of control patients, GR type, total number of sites, number of test sites, number of control sites, primary and secondary outcomes of each study, and patient‐reported outcome test (pain, hypersensitivity, esthetic, satisfaction).

### 2.7 Outcome Variables

The variables sought in each study were defined as follows:

○ Complete root coverage (CRC) is the percentage of sites that obtained complete radicular coverage at a given time of follow‐up, with respect to the total number of treated sites. CRC = (no. of sites with CRC)/(total n. of sites treated) × 100%;
○ Percentage of Root Coverage (%RC) or Mean Root Coverage (MRC%), which describes the rate of recession reduction compared to the baseline recession value;
○ Recession reduction (RecRed), it is the difference (mm) between the recession measured at a given follow‐up and baseline recession value.
○ The differential clinical attachment level (ΔCAL) reflects the gain of CAL (mm) at a given follow‐up.
○ Differential keratinized tissue width (ΔKTW). Differences between follow‐up and baseline values. The KTW is the distance (mm) from the free gingival margin to the mucogingival junction.
○ Differential keratinized tissue thickness (ΔKTt or ΔGT). KTt and GT indicate the thickness of the attached gingiva (mm).
○ Postoperative pain (VAS), which is a score ranging from 0 (no pain at all) to 100 (worst imaginable pain) reported daily by the patient until the seventh day after the surgery.
○ Duration of the surgery in minutes from the first incision to the last suture.

### 2.8 Evaluation of Risk of Bias

The risk of bias of the included RCTs was assessed using the Cochrane Collaboration tool. The following parameters were considered: random sequence generation and allocation concealment (selection bias); blinding of participants and personnel (performance bias); blinding of outcome assessment (detection bias); incomplete outcome data (attrition bias); selective reporting (reporting bias); and other possible reasons for bias.

### 2.9 Meta‐Analysis

For each continuous variable, the effects were measured using the mean difference, and an inverse variance statistical method was used. The study confidence interval and the total confidence interval were set at 95%. Meta‐analysis was performed using the Review Manager 5.4 software (RevMan 5.4.1, The Cochrane Collaboration, 2020). The heterogeneity among the studies' effects was evaluated using the Q Cochrane test, relative *p* values, and *I* ^2^ statistics. When the heterogeneity was small (*I* ^2^ < 60%, *p* > 0.05), a fixed effects model was used. Otherwise, a random effects model analysis was performed. Parallel and split‐mouth studies were combined in a meta‐analysis of treatment effects. A meta‐analysis was performed when at least three included studies reported the same outcomes, the data of which could be combined. Recession was used as the analysis unit. The level of significance was set at *p* = 0.05.

## 3 Results

### 3.1 Study Selection

**FIGURE 1:**
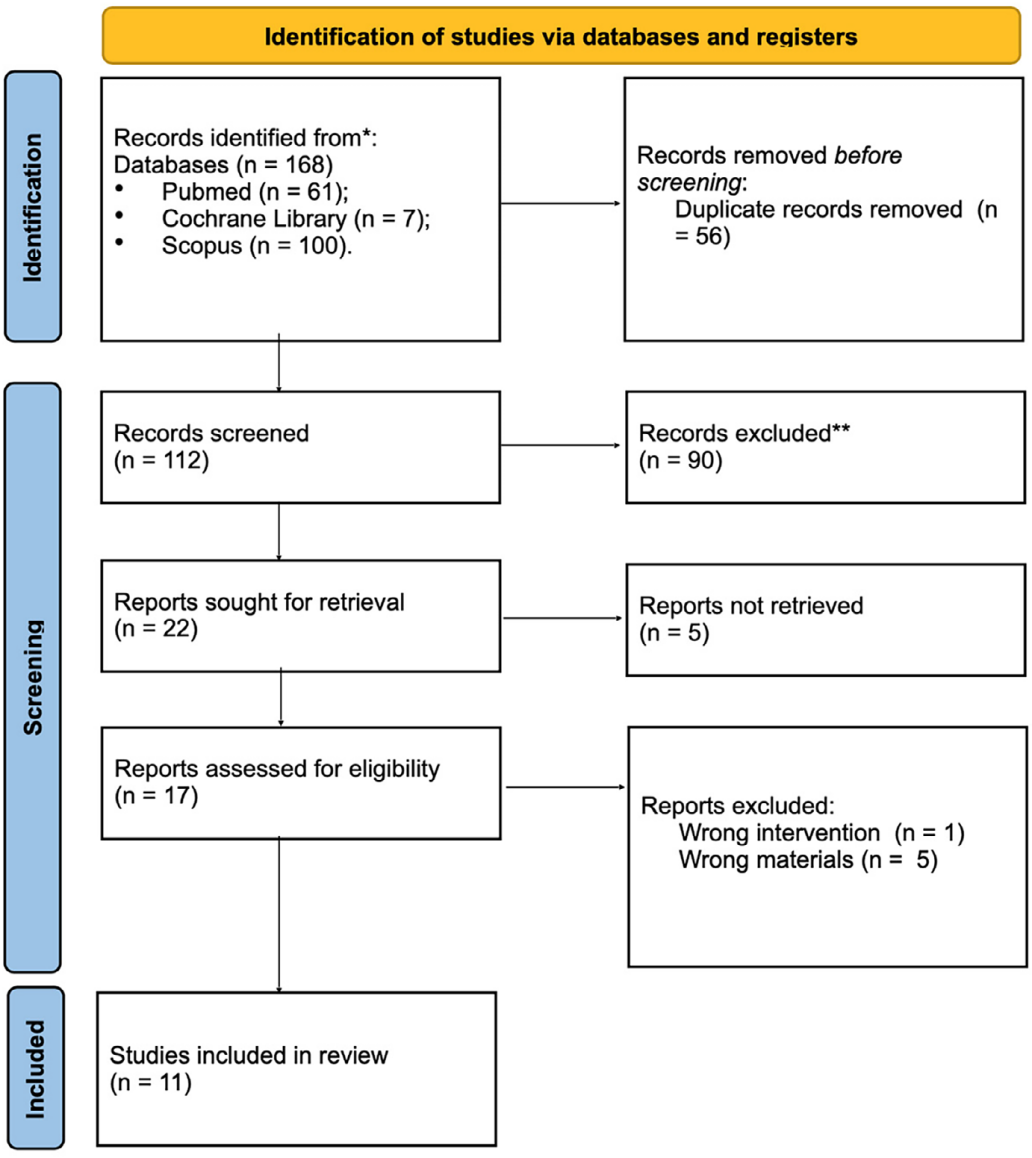
PRISMA flowchart.

The electronic search through the PubMed database identified 61 publications, the search using the Cochrane Library database identified seven titles (trials), and the search using the Scopus database identified 100 articles (a total of 168 articles). After removing all duplicates, 112 articles were identified from 2013 to 2023. After reading all titles and abstracts, 22 studies were retrieved, and for 17 studies positive for eligibility, the full text was obtained. After full‐text reading, six articles were excluded (five due to ineligible materials and one due to ineligible intervention). Finally, 11 studies were included (Aroca et al. 2013; McGuire and Scheyer 2016; Moreira et al. 2016; Tatarakis et al. 2018; Tonetti et al. 2018, 2021; Rotundo et al. 2019; Barakat and Dayoub 2020; Nahas et al. 2020; Molnár et al. 2022; Lakshmi et al. 2023). The main features of the included studies are presented in Table 1. A flow chart summarizing the study selection procedure in accordance with PRISMA guidelines (2020) is presented in Figure 1.

**TABLE 1:** Characteristics of the included studies.

Authors	Year	Design	No Patients	No Sites	Test surgery	Control surgery	Type of defect	Follow up (months/years)
Aroca et al. (1)	2013	RCT (split mouth)	22	156	mCAT + CMX	mCAT + sCTG	Multiple GRs	12 months
McGuire et al. (2)	2016	RCT (split mouth)	17	34	CAF + CMX	CAF + sCTG	Single GR	5 years
Moreira et al. (3)	2016	RCT (parallel groups)	40	40	CAF + CMX	CAF	Single GR	6 months
Tatarakis et al. (4)	2018	RCT (parallel groups)	8	8	CAF + CMX	CAF + sCTG	Single GR	6 months
Tonetti et al. (5)	2018	RCT (parallel groups)	187	485	CAF + CMX	CAF + sCTG	Multiple GRs	6 months
Rotundo et al. (6)	2019	RCT (parallel groups)	24	61	CAF + CMX	CAF	Multiple GRs	12 months
Barakat et al. (7)	2020	RCT (split mouth)	22	44	CAF + CMX	CAF + sCTG	Single GR	12 months
Nahas et al. (8)	2020	RCT (split mouth)	15	82	mCAF + CMX	mCAF + sCTG	Multiple GRs	12 months
Tonetti et al. (9)	2021	RCT (parallel groups)	89	219	CAF + CMX	CAF + sCTG	Multiple GRs	3 years
Molnár et al. (10)	2022	RCT (split mouth)	16	114	mCAT + CMX	mCAT + sCTG	Multiple GRs	9 years
Lakshmi et al. (11)	2023	RCT (split mouth)	28	64	mCAT + CMX	mCAT + sCTG	Multiple GRs	6 months

Abbreviations: CAF, coronally advanced flap; CMX, collagen matrix; GR, gingival recession; mCAF, modified coronally advanced flap; mCAT, modified coronally advanced tunnel; RCT, randomized controlled trial; sCTG, subepithelial connective tissue graft.

### 3.2 Risk of Bias

**FIGURE 2:**
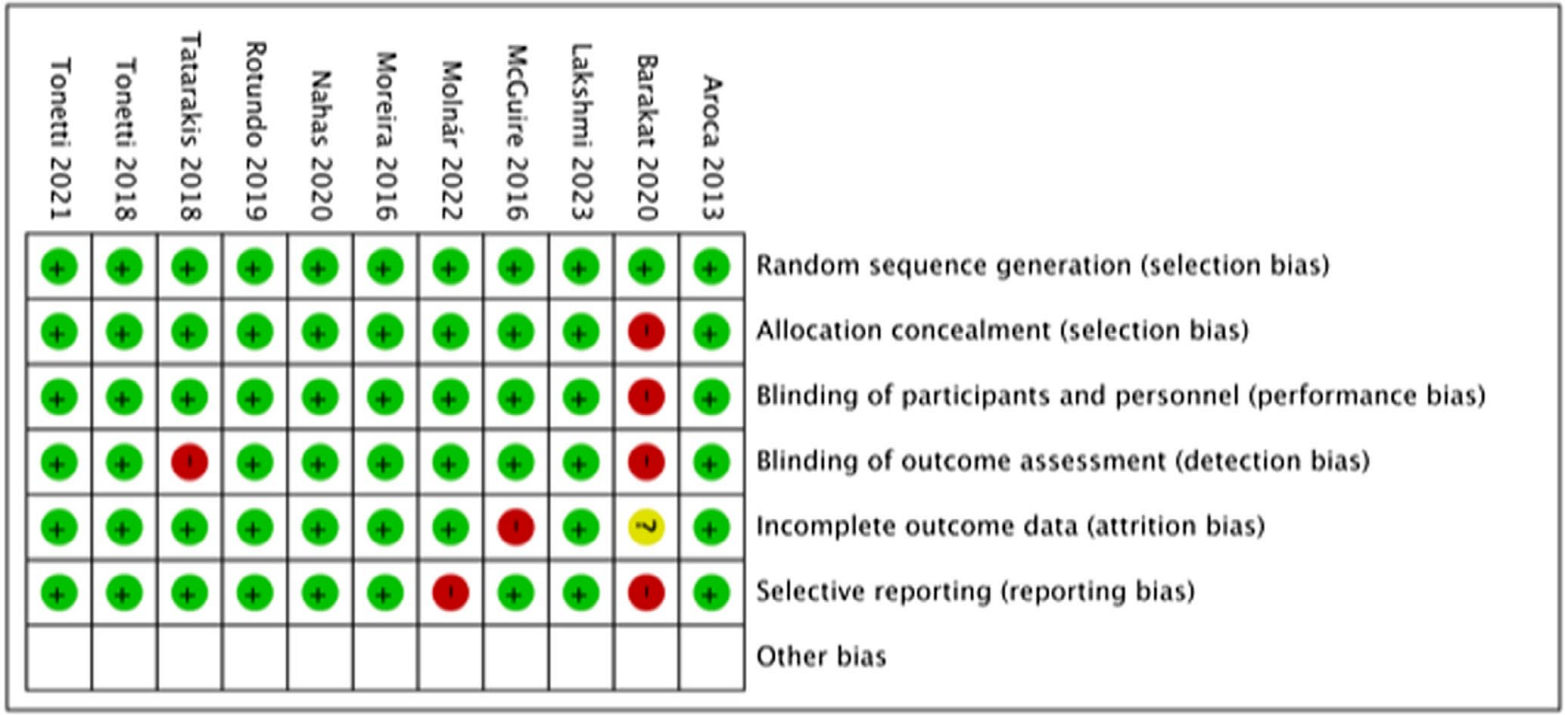
Risk of bias summary.

In the first analyzed parameter, Random sequence generation, every study satisfied the criteria to be classified as having a low risk of bias. For the second and the third parameter, the allocation concealment and blinding of participants and personnel, every study was classified at low risk of bias, except for one study (Barakat and Dayoub 2020) which was classified at high risk of bias because there was no evidence in the paper about the allocation concealment and blinding of participants and personnel protocol. Regarding the fourth parameter, blinding of outcome assessment, every study was classified as having a low risk of bias, except for two studies (Barakat and Dayoub 2020; Tatarakis et al. 2018), which were classified as having a high risk of bias because there was no evidence of blinding of the evaluators. In one study (McGuire and Scheyer 2016), the fifth parameter, Incomplete outcome data, was categorized as high risk of bias because of the original 25 patients; 17 were available for a 5‐year recall. Seven of the eight patients unavailable for recall had moved, were not reachable, or had conflicting engagements, and one had received a class 5 restoration that eradicated the baseline measurement reference point. The percentage of withdrawals and dropouts should not exceed 30% during long term follow‐up. Other studies were classified as low‐risk for the fifth parameter. One study (Barakat and Dayoub 2020) was considered to have an unclear risk of bias for *Selective* reporting bias, whereas the other studies were considered at low risk of bias. Overall, seven studies were judged to have a low risk of bias, and four were at high risk (Figure 2).

### 3.3 Results of Individual Studies

CRC was reported in nine articles and ranged from 9.01% to 88.2%. The %RC has been reported in eight articles. RecRed and ΔKTw have been reported in six articles. ΔCAL and ΔPD were reported in four studies. The ΔKTt was reported in three studies. The duration of surgery was reported in five articles. Post‐surgical pain (VAS) was reported in four articles. No study has reported the complete root coverage %, differential CAL, or VAS score.

### 3.4 Synthesis of Results

To reduce the heterogeneity between studies and improve the quality of the review, only studies with a minimum of six‐month follow‐up were included. Eleven publications were included in the meta‐analysis (Aroca et al. 2013; McGuire and Scheyer 2016; Moreira et al. 2016; Tatarakis et al. 2018; Tonetti et al. 2018, 2021; Rotundo et al. 2019; Barakat and Dayoub 2020; Nahas et al. 2020; Molnár et al. 2022; Lakshmi et al. 2023), and five parameters were considered: root coverage percentage (%RC), reduction of recession (REC reduction), differential keratinized tissue width (ΔKTw), differential keratinized tissue thickness (ΔKTt), and surgery duration. Each parameter was assessed by distinguishing single from multiple recessions and for each parameter, a forest plot was created to explore the technique that could lead to better results. The results of the comparisons are presented in the following paragraphs.

### 3.5 Meta‐Analysis Results

#### 3.5.1 Root Coverage Percentage (%RC)

**FIGURE 3:**
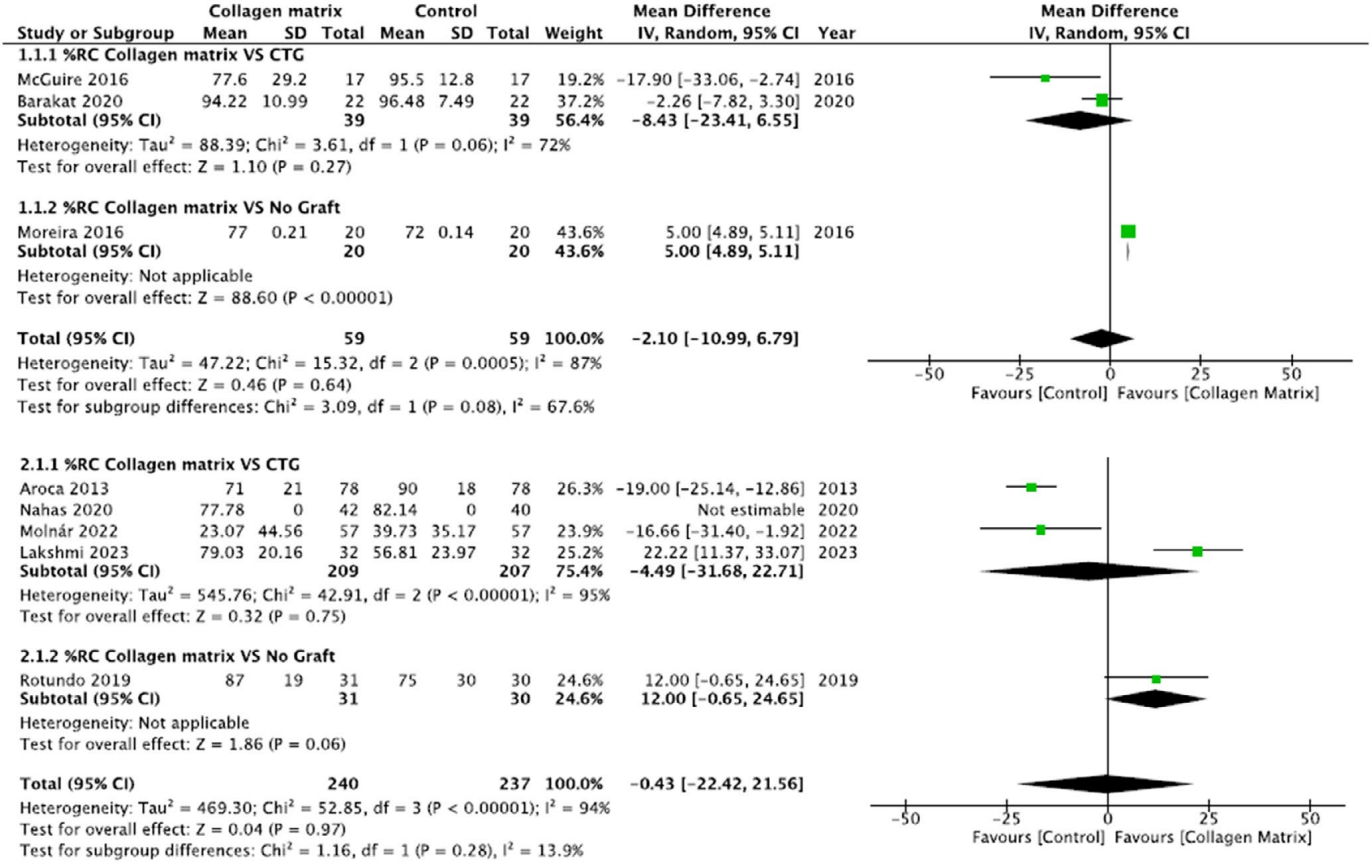
Forest plot of %RC (root coverage). Upper panel: Single recession; Lower panel: Multiple recession—Comparison: Collagen matrix vs. CTG or Collagen matrix vs. No Graft.

It was decided to split the analysis by introducing two different subgroups: one comparing CAF/mCAT + sCTG vs. CAF/mCAT + CMX, and one comparing CAF without any grafting material vs. CAF/mCAT + CMX. Analyzing single recessions, two articles were included in the first subgroup (McGuire and Scheyer 2016; Barakat and Dayoub 2020) and one in the second subgroup (Moreira et al. 2016). In the multiple recessions group, four studies were included in the first subgroup meta‐analysis (Aroca et al. 2013; Lakshmi et al. 2023; Molnár et al. 2022; Nahas et al. 2020), and only one in the second subgroup (Rotundo et al. 2019). One study (Nahas et al. 2020) was excluded from statistical analysis because no standard deviations were present in the original article. Given the considerable heterogeneity, a random effects model was used (Figure 3).

In the single recession group, 20 sites were treated with CAF/mCAT + CMX and CAF/mCAT + sCTG, respectively. Despite the high prevalence of results in favor of CAF/MCAT + SCTG, the difference between the two groups of intervention was not statistically significant (*p* = 0.27). Comparing the CAF technique employing a CMX (20 sites) with the CAF alone (20 sites), the meta‐analysis showed better, statistically significant results (*p* < 0.00001), in favor of CMX instead of not using it.

In the multiple recessions group, a total of 209 and 207 sites were treated with CAF/mCAT + CMX and CAF/mCAT + sCTG, respectively. Despite the high prevalence of results in favor of CAF/MCAT+SCTG, the difference between the two groups of intervention was not statistically significant (*p* = 0.75). Comparing the CAF technique employing a CMX (31 sites) with the CAF alone (30 sites), the meta‐analysis showed better and close to statistical significance results (*p* = 0.06), in favor of CMX instead of not using it.

#### 3.5.2 REC Reduction

In the single recession group, for the analysis of the recession reduction (RecRed), one study was considered (Barakat and Dayoub 2020) in the first subgroup (CAF/mCAT + sCTG vs. CAF/mCAT + CMX) and one (Moreira et al. 2016) in the second (CAF without any grafting material vs. CAF/mCAT + CMX).

**FIGURE 4:**
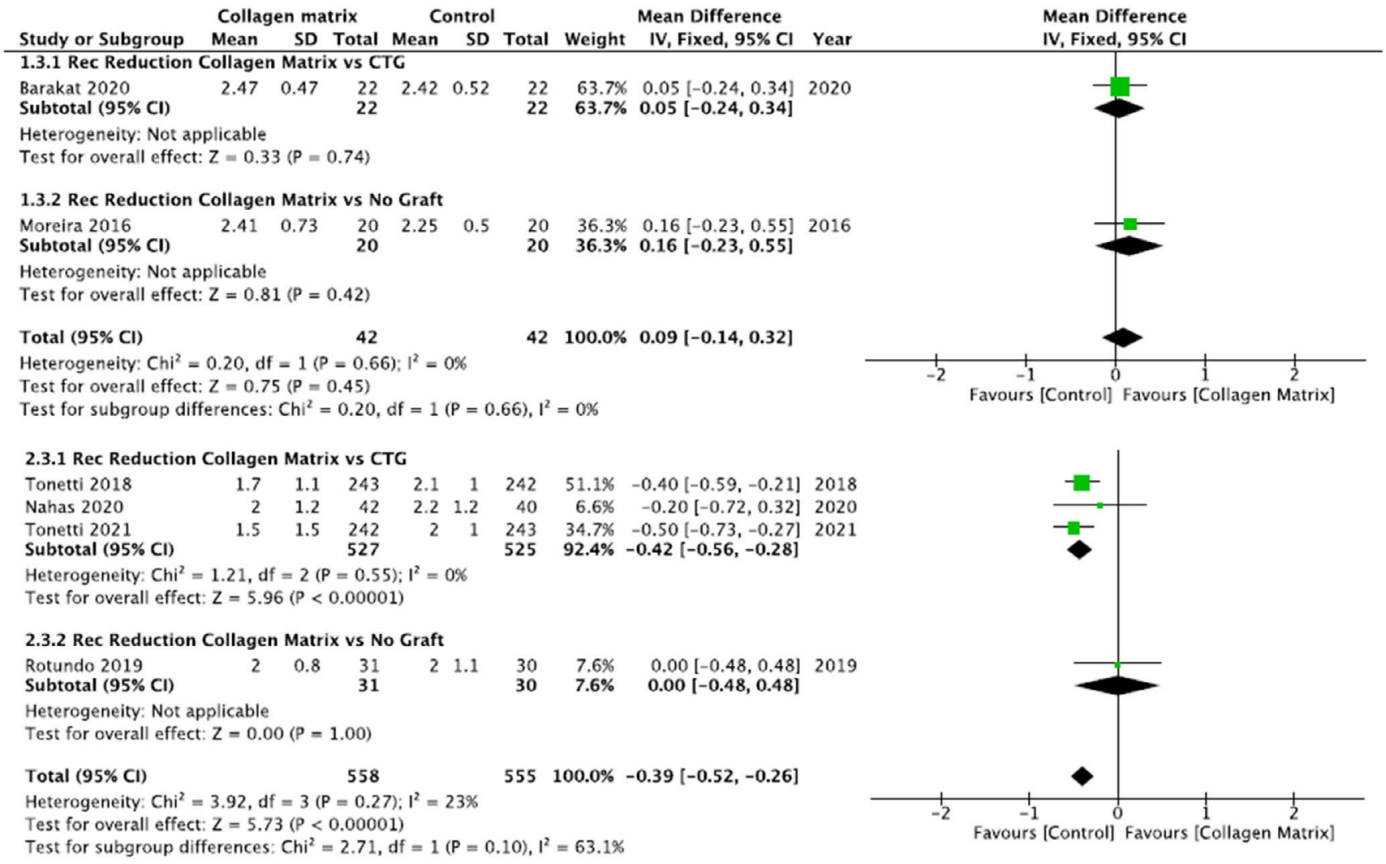
Forest plot of recession reduction. Upper panel: Single recession; Lower panel: Multiple recessions—Comparisons: Collagen matrix versus. CTG or Collagen matrix versus No Graft.

For the multiple recession group, three studies were included in the first subgroup (Nahas et al. 2020; Tonetti et al. 2018, 2021) and only one (Rotundo et al. 2019) in the second subgroup (Figure 4).

In the single recession group, 22 sites were treated with CAF/mCAT + CMX and CAF/mCAT + sCTG, respectively and there wasn't a statistically significant difference between experimental and control groups (*p* = 0.74). Also in the second subgroup, which compared REC reduction in 20 sites treated with CAF/mCAT + CMX (test group) and 20 treated with CAF alone, there was no statistically significant difference between the two groups (*p* = 0.42).

In the multiple recessions group, 527 sites were treated with CAF/mCAT + CMX and 525 sites with CAF/mCAT + sCTG, respectively and there was a statistically significant difference between experimental and control groups in favor of the CAF/mCAT + sCTG group (*p* < 0.00001).

Comparing the CAF technique employing a CMX (31 sites) with the CAF alone (30 sites), the meta‐analysis showed no differences between the two groups (*p* = 1.00).

#### 3.5.3 ΔKTw

In the single recession group, it was possible to evaluate differential keratinized tissue width (ΔKTw) only in one study (Barakat and Dayoub 2020), while for the multiple recession group, four studies were included (Molnár et al. 2022; Nahas et al. 2020; Tonetti et al. 2021, 2018) in the first subgroup and one in the second.

**FIGURE 5:**
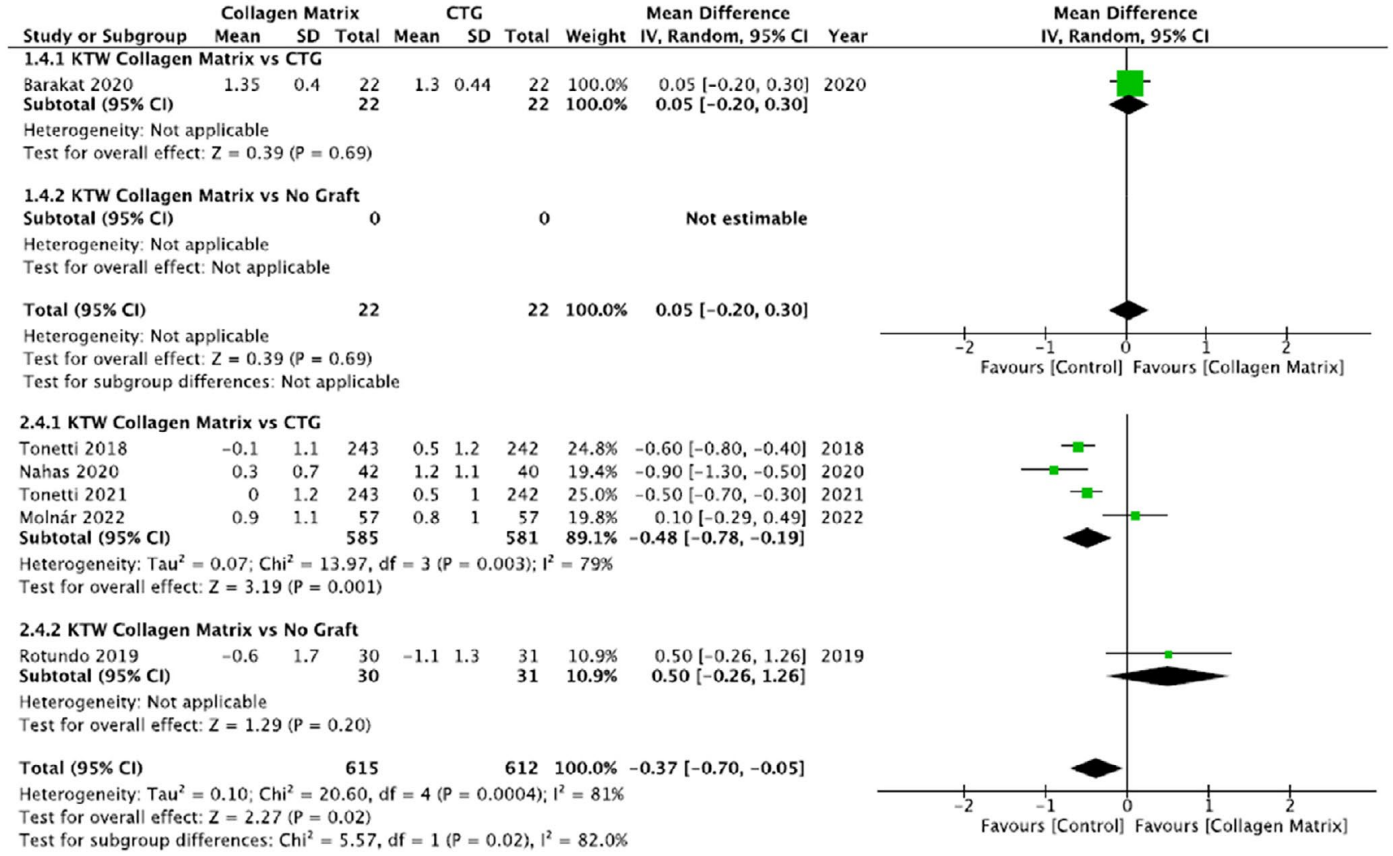
Forest plot of ΔKTw. Upper panel: Single recession; Lower panel: Multiple recessions—comparisons: Collagen matrix versus. CTG or Collagen matrix versus No Graft.

For the single recession group, none of the selected studies presented data about the ΔKTw comparing CMX and CAF alone (Figure 5).

For the single recession group, no statistical difference came up from the meta‐analysis of 22 sites in the test group (mCAT/CAF + CMX) and in the control group (mCAT/CAF + sCTG) (*p* = 0.69).

In the multiple recession group, the meta‐analysis of 585 patients in the test group (mCAT/CAF + CMX) and 581 patients in the control group (mCAT/CAF + sCTG) showed a significant difference in ΔKTw between the two groups in favor of the control group (*p* = 0.001). In the second subgroup a statistically significant difference was observed (*p* = 0.02) in terms of ΔKTw by comparing CMX and CAF alone in multiple recession treatment.

#### 3.5.4 ΔKTt

In the single recession group, it was possible to evaluate differential keratinized tissue thickness (ΔKTt) only in one study (Moreira et al. 2016), which compare CMX and CAF alone. None of the selected studies presented data about the ΔKTt comparing mCAT/CAF + sCTG and mCAT/CAF + CMX in single recession treatment.

**FIGURE 6:**
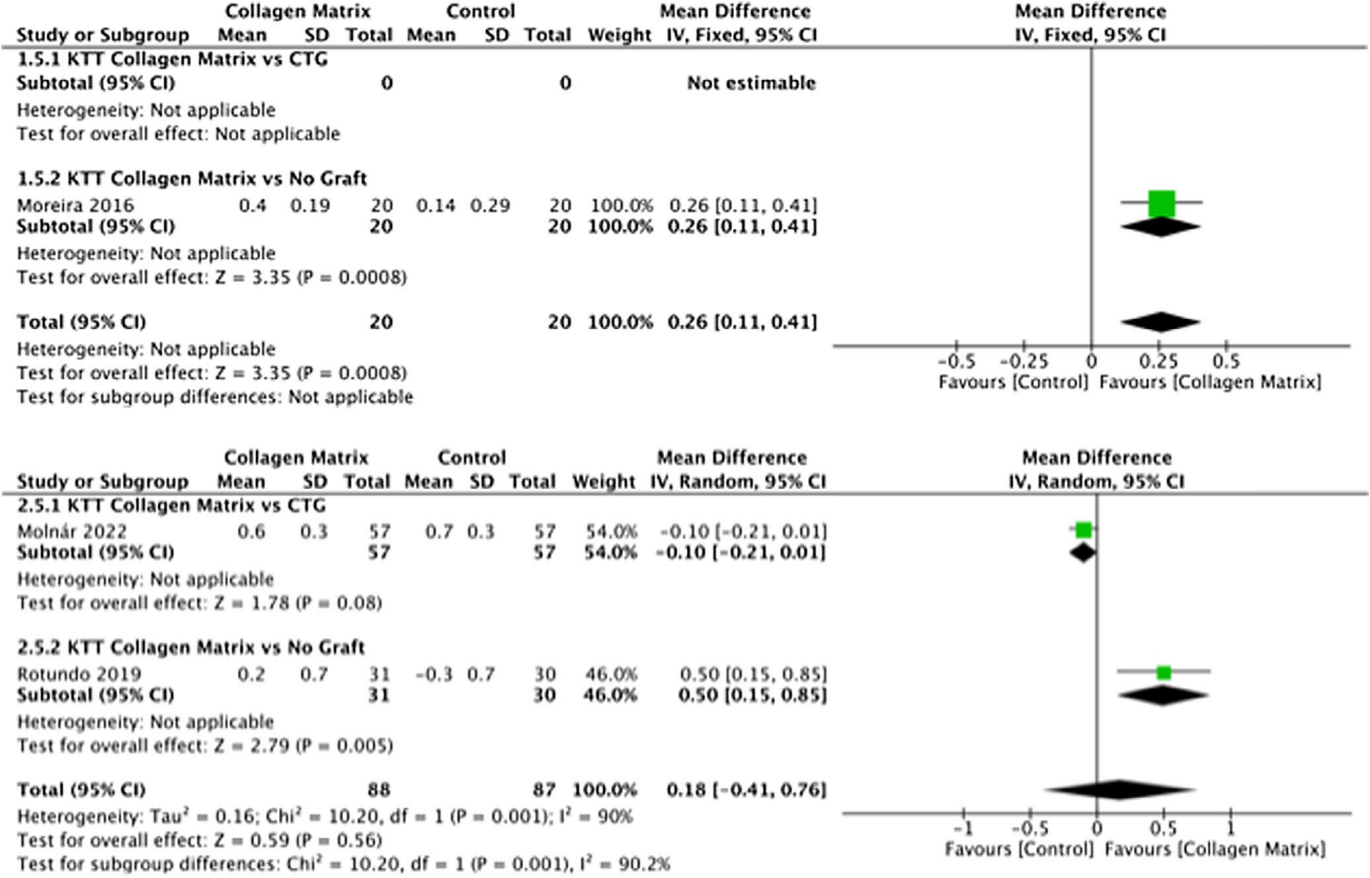
Forest plot of ΔKTt. Upper panel: Single recession; Lower panel: Multiple recessions—comparisons: Collagen matrix versus. CTG or Collagen matrix versus No Graft.

In the multiple recessions group, one (Molnár et al. 2022) was considered in the first subgroup and one study in the second subgroup (Rotundo et al. 2019) (Figure 6).

In this meta‐analysis, there was a statistically significant difference between the two techniques in the single recession group in favor of CAF + CMX compared with CAF alone in terms of ΔKTt (*p* < 0.0008).

Comparing mCAT/CAF + sCTG and mCAT/CAF + CMX in the multiple recession group, there was a difference close to statistical significance in favor of mCAT/CAF + sCTG (*p* = 0.08) while the statistical difference founded comparing CAF + CMX with CAF was significant in favor of CAF + CMX (*p* = 0.005).

#### 3.5.5 Duration of Intervention

In the single recession group, for the analysis of the time of surgery, one study was considered (Barakat and Dayoub 2020) in the first subgroup (CAF/mCAT + sCTG vs. CAF/mCAT + CMX). It wasn't possible to perform a statistical analysis comparing CAF + CMX and CAF alone for the single recession treatments for lack of data.

**FIGURE 7:**
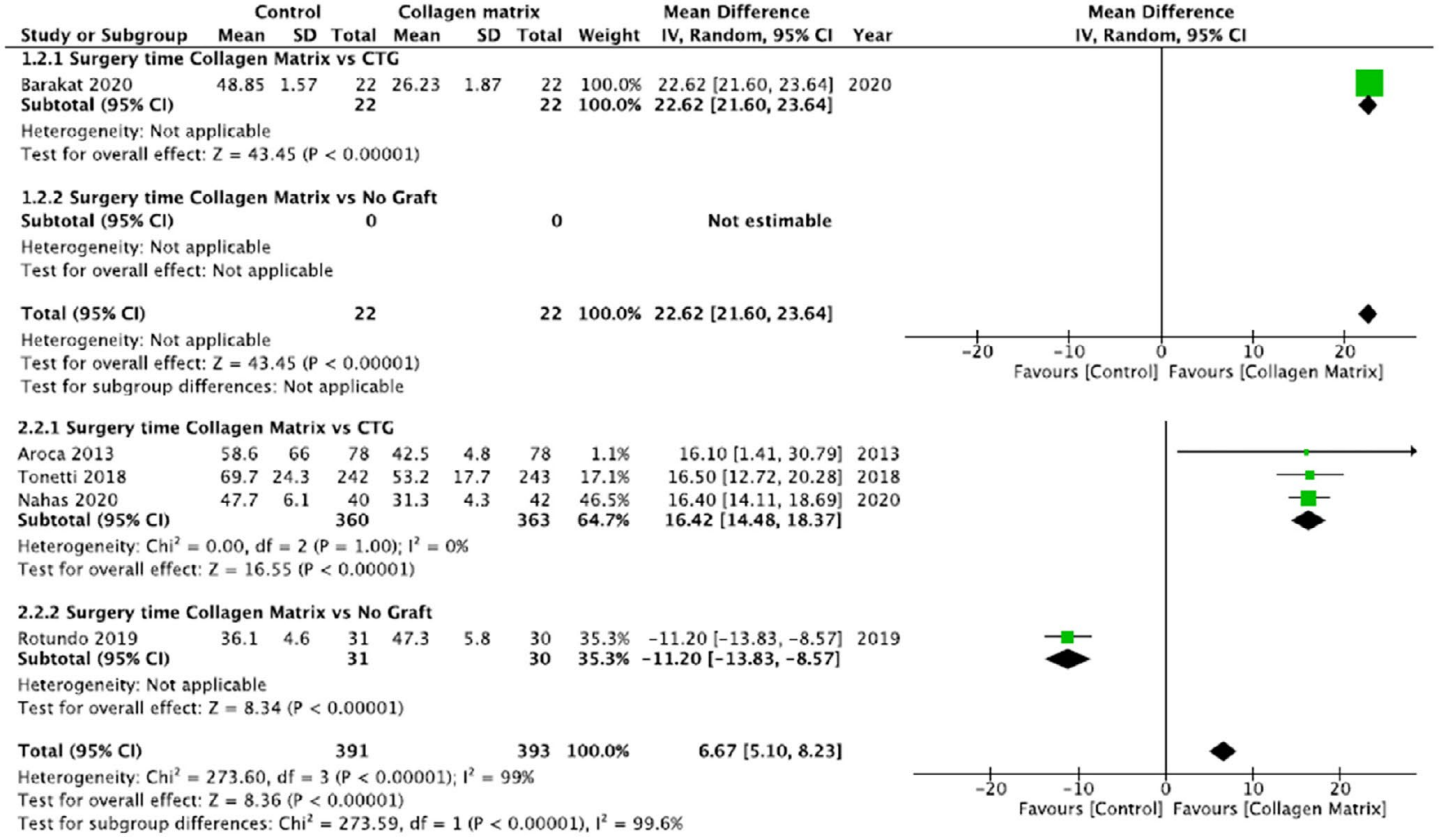
Forest plot of Surgery duration. Upper panel: Single recession; Lower panel: Multiple recessions. Comparisons: Collagen matrix versus. CTG or Collagen matrix versus No Graft.

In the multiple recessions group, three studies were analyzed in the first subgroup (Aroca et al. 2013; Nahas et al. 2020; Tonetti et al. 2018) and only one study in the second (Rotundo et al. 2019) (Figure 7).

The meta‐analysis revealed a significant decrease in the duration of surgery in the group of mCAT/CAF + CMX respect to the mCAT/CAF + sCTG group (*p* < 0.00001) in the single recession group as well as in the multiple recession one (*p* < 0.00001). Moreover, it shows a significant difference in favor of CMX group compared with CAF alone (*p* < 0.00001).

## 4 Discussion

The demand for recession treatment in recent years has significantly increased because of both esthetic needs and the increasing number of post‐orthodontic recessions, especially in young patients (Jati, Furquim, and Consolaro 2016; Slutzkey and Levin 2008; Bin Bahar et al. 2020). The Coronally Advanced Flap is considered by the scientific community of periodontologists as one of the most effective and safe techniques for treating single and multiple recessions (Toledano‐Osorio et al. 2022). Numerous studies have shown that the use of a subepithelial connective tissue graft can enhance this technique (Carvalho et al. 2006; Chambrone and Tatakis 2015; Cairo, Pagliaro, and Nieri 2008), stabilizing results and thickening tissues in the medium‐ and long‐term, especially in thin phenotypes (Cairo et al. 2016). In recent years, alternative techniques have been proposed and seem to provide, in expert hands, results similar to those obtained with the CAF, such as the Coronally Advanced Tunnel (CAT) technique (Zabalegui et al. 1999; González‐Febles et al. 2023) and the latest modified version mCAT (Azzi et al. 2002; Zuhr et al. 2007; Stähli et al. 2023; Molnár et al. 2022). Subepithelial connective tissue grafts can also be used with these techniques to maximize the outcome (Nart and Valles 2016; Santamaria et al. 2023; Pini Prato et al. 2018). The harvesting of connective tissue today is therefore a fundamental weapon for this type of intervention, as it may produce excellent results but has some disadvantages such as a secondary site of intervention, lengthening of the operating time, increased postoperative morbidity, and pain. In recent years, companies have tried to produce materials that can mimic the features of connective tissue, thus eliminating the complications associated with harvesting it from the palate. Collagen‐derived matrices have been introduced to satisfy the requirements of reduced operator time and patient discomfort. In this systematic review, we analyzed 11 studies (Rotundo et al. 2019; Aroca et al. 2013; Nahas et al. 2020; Barakat and Dayoub 2020; Tonetti et al. 2018, 2021; McGuire and Scheyer 2016; Tatarakis et al. 2018; Moreira et al. 2016; Lakshmi et al. 2023; Molnár et al. 2022) in which subepithelial connective tissue or CAF alone were compared to CMXs.

### 4.1 % Root Coverage (RC)

The meta‐analysis for %RC did not identify a statistically significant difference by using SCTG instead of CMX in single recession (*p* = 0.27) and multiple recession (0.75) groups, even if it seems that the use of sCGT can lead to a clinically better result in terms of %RC. One study (Lakshmi et al. 2023) found contrasting results when compared with the others. This RCT evaluated smokers (≥ 10 cigarettes/day for ≥ 5 years), suggesting that in this type of patient, CMX might even work better than connective tissue. Nevertheless, a recent systematic review (Moscowchi et al. 2023) showed a significant difference at 6 months in terms of CRC comparing non‐smokers and individuals who smoked 10–20 cigarettes/day, in favor of non‐smokers, OR = 0.15 (95% CI = 0.03 to 0.71; *p* = 0.017).

Comparing CMX to CAF alone, the difference in %RC between the two groups was statistically significant (*p* < 0.0001) in the single recession group and close to statistical significance in the multiple recession group (*p* = 0.06), suggesting that the use of CMX may lead to better %RC than no grafting.

### 4.2 Recession Reduction

In the meta‐analysis of recession reduction, there was weak evidence of a difference between the two groups for the single recessions (*p* = 0.74); however, by considering the multiple recessions, there was a strong trend for SCTG to provide better results in terms of recession reduction (*p* < 0.00001). In single recession treatment, the use of CMX seems to achieve slightly better recession reduction compared with CAF without any graft (*p* = 0.42), while in the multiple recession treatment, the use of CMX seems to not bring any kind of vantage in terms of Recession reduction (*p* = 1).

### 4.3 Difference in Keratinized Tissue Width (ΔKTw) and Thickness (ΔKTt)

The meta‐analysis of ΔKTw showed that by using sCTG rather than CMX in single recession treatment, there is not a statistically significant advantage in terms of keratinized tissue width (*p* = 0.69) on the contrary it seem that CMX provides slightly better result than sCTG. Conversely, in multiple recessions treatment, the use of sCTG led to a better and statistically significant results in ΔKTw (*p* = 0.001) if compared to CMX.

The use of sCTG seems to increase the thickness of keratinized tissue (ΔKTt) when compared with CMX (*p* = 0.08) in multiple recessions. On the other hand, CMX provides way better results in both ΔKTw and (ΔKTt) when compared with CAF alone.

### 4.4 Duration of the Intervention

Regarding the duration of the intervention, the meta‐analysis showed a statistically significant advantage in the use of CMX when compared with sCTG in both single and multiple recessions (*p* < 0.00001). This result is not surprising if we consider that harvesting the connective tissue from the palate and suturing takes much more time than ready‐for‐use CMX. The difference in timing could be greater depending on the technique used for harvesting the tissue or if we need to manage intraoperative bleeding from the donor site.

Meta‐analysis of post‐surgery pain (VAS) was not possible because of the wide heterogeneity of data among studies. One study (Rotundo et al. 2019) showed a significant difference in pain perception using a VAS scale at day 1 between the CAF group (1.5 ± 1.9) and the CAF + CMX group (2.7 ± 2.7) in favor of the first one, but at day 6, the pain perception was inverted within the two groups (0.4 ± 0.9 and 0.2 ± 0.4). Another study (Tatarakis et al. 2018) reported an initial post‐surgery pain at day 1 for the CAF + sCTG group (43 ± 25.59) double compared to CAF + CMX (22.5 ± 12). On day 14, the perception of pain in the CAF + CMX group was twice as high as that in the CAF + sCTG group. The trend reversal recorded in these two studies might suggest that after 1 week, the sCTG could be better accepted than the CMX, which could trigger more inflammation during its integration.

In contrast, another two studies (Nahas et al. 2020) documented increased values in terms of VAS for the mCAF+sCTG group compared to the mCAF+CMX group without any kind of trend reversal, which underlined the divergence and subjective data about post‐surgery pain.

### 4.5 Esthetic Outcomes

One of the main aims of surgical recession coverage is to achieve satisfactory esthetic outcomes.

In one study (Nahas et al. 2020) the esthetic result, measured on a VAS scale from 0 to 10, reported similar findings between the two groups: at 1 year 9.57 in the sCTG group and 9.23 in the CM group at the patient level, and 8.94 and 8.40 at the professional level. In both groups, patient and professional, the differences in the esthetic outcomes between the two techniques were not statistically significant (*p* = 0.637 and *p* = 0.834). In another study (Aroca et al. 2013) the esthetic result at one‐year follow‐up was also based on a VAS scale: similar results were found in the two groups with a slight trend in favor of CMX (92.9 ± 8.4) compared to sCTG (90.6 ± 7.9). Rotundo et al. (2019) reported no substantial differences between the two groups CAF + CMX and CAF alone.

In McGuire and Scheyer study (McGuire and Scheyer 2016), they found that there were no significant differences comparing CMX + CAF and sCTG + CAF in terms of color match to surrounding tissues at 6 months (*p* > 0.99) and 5 years (*p* = 0.63) follow‐up. On the contrary, there was a statistically significant difference between the two techniques in favor of CAF + sCTG by analyzing the texture at 6 months (*p* = 0.006) and 5 years (*p* = 0.002). Nevertheless, patient satisfaction was high and similar within the two groups at 6 months and 5 years (*p* = 0.13 and *p* > 0.99). Also, the study of Moreira et al. (2016) reported an overall patient satisfaction greater than 90 with no differences within the two groups. Moreover, the study of Barakat and Dayoub (2020) reported similar results in patient esthetic satisfaction with no significant differences within the two groups.

The study of Lakshmi et al. (2023) showed values of RCES higher for the CMX group (8.19 ± 1.70) than for the sCTG group (6.36 ± 1.81) with a statistical significance (*p* < 0.001), not according with a recent paper of (Pelekos et al. 2019) in which the authors used the RES (Root Esthetic Score) to assess the esthetic result after root coverage of multiple adjacent recessions with CAF + CMX and CAF + sCTG. They found a better overall RES score for the sCTG group, but on the other hand a better marginal tissue texture and marginal contour were observed in the CMX group.

### 4.6 % Complete Root Coverage (CRC)

The use of CMX provided better results in terms of %CRC using CAF/mCAF + CMX technique (54.5 mean %CRC) rather than mCAT+CMX (33.8%CRC mean).

One study (Tonetti et al. 2018) concluded that there are unquestionable benefits for the patient derived from the use of CMX instead of sCTG in multiple adjacent recessions. However, these matrices still show inferior results compared to sCTG. Nahas et al. (2020) affirmed that the results between the two groups were quite similar at 1 year, as well as the authors of two other studies (Barakat and Dayoub 2020; McGuire and Scheyer 2016), who suggested that the result obtained by using CAF + CMX in recession covering at 5 years follow‐up could be retained, even if inferior to the stability of sCTG. One study (Moreira et al. 2016) compared the CAF + CMX to the CAF alone and concluded that both may lead to good results in gingival recession coverage and the CAF + CMX. Even if it does not provide better outcomes in the recession reduction after 6 months, it slightly increases tissue thickness. Also, another study (Rotundo et al. 2019) reported that by comparing CAF + CMX to CAF alone, the results after 1 year were quite similar, also regarding the patient‐reported outcomes. Furthermore, the use of CMX in association with CAF significantly increased gingival thickness. One study (Aroca et al. 2013) concluded that, due to the reduction of surgical times and patient morbidity, the use of CMX is a good alternative, but the use of sCTG in association with mCAT generally provides better results in the treatment of Miller Class I and II. Interestingly, in one study (Tatarakis et al. 2018) it was observed that the flow alterations in the early healing of the connective tissue graft and collagen matrices followed similar patterns. CMX showed a secondary increase in blood flow, probably owing to its remodeling properties. If, on the one hand, the connective tissue showed better results in terms of root coverage and keratinized tissue gain, on the other hand, the use of the CMX was associated with a lower initial morbidity.

One study (Lakshmi et al. 2023) suggested that the use of mCAT+CMX could be considered as a valid alternative compared to mCAT + sCTG especially for smokers.

Another study (Molnár et al. 2022) concluded his long term study affirming that both MCAT with SCTG and CMX are subject to deterioration within 9 years, and the results obtained in the maxillary sites are generally more stable than the mandibular ones.

A study by Tonetti et al. (2021) concluded that CAF + sCTG is probably the best approach for the treatment of multiple adjacent recessions, as also supported by their previous study (Tonetti et al. 2018), but the avoidance of the donor site using CMX resulted in shorter recovery times and less post‐operative morbidity, as suggested by other authors (Pelekos et al. 2019), in a more natural soft tissue texture and contour Tonetti et al. (2021), comparing CAF + sCTG with CAF + CMX showed similar results in terms of stability between 6 and 36 months.

The main limitations of this review are that the treated recessions have not been stratified according to the severity class RT and that single recessions have not been diversified from multiple recessions.

## 5 Conclusions

Based on the available data, it was determined that surgical treatment for recession coverage performed with collagen derivatives such as Mucograft (Geistlich Pharma) allows for good clinical outcomes in the short and medium term when compared to the gold standard, sCTG. When compared with CAF alone, CMX provides similar results in terms of root coverage but undoubtedly better results in increasing gingival thickness. Compared to sCTG, it has the advantage of not requiring a second site of intervention and reducing the operating time; however, in our opinion, it can only provide satisfactory results in Miller Classes I and II, while sCTG is still preferred in more complex cases. The indications for using one surgical approach rather than another are different and depend a great deal on the starting conditions, especially the gingival phenotype, the thickness of the keratinized tissue, its width, and, not least, the type of recession to be treated. Nevertheless, the use of collagen matrices can provide undoubted advantages such as avoiding a second surgical site, thus decreasing post‐operative discomfort as well as surgery time, or treating numerous recessions that require improving tissue quality with a graft in a single surgery. Moreover, CMX seems to provide good esthetic results, sometimes better than sCTG, especially when employed to treat thin phenotypes, as suggested by one study (Rotundo et al. 2019), but its long‐term root coverage stability performance is still uncertain.

## Author Contributions

**Alessandro Zangani:** conceptualization. **Miriana Gualtieri:** conceptualization. **Alessia Pardo:** conceptualization, data curation, formal analysis, writing – original draft, writing – review and editing, validation, software. **Annarita Signoriello:** writing – review and editing, data curation. **Paolo Faccioni:** data curation, investigation, validation. **Gianluca Colapinto:** methodology, conceptualization, writing – original draft, validation, writing – review and editing, visualization. **Funda Goker:** writing – original draft, methodology, writing – review and editing, validation. **Giorgio Lombardo:** data curation, formal analysis, writing – review and editing. **Massimo Del Fabbro:** writing – review and editing, data curation, conceptualization, writing – original draft, validation, methodology, supervision, visualization. **Massimo Albanese:** resources, validation, writing – review and editing, project administration, supervision, visualization, funding acquisition.

## Ethics Statement

The authors have nothing to report.

## Consent

The authors have nothing to report.

## Conflicts of Interest

The authors declare no conflicts of interest.

## Data Availability

The data that support the findings of the study are available at the corresponding authors upon reasonable request.
